# Novel CLC3 transcript variants in blood eosinophils and increased CLC3 expression in nasal lavage and blood eosinophils of asthmatics

**DOI:** 10.1002/iid3.36

**Published:** 2014-12-04

**Authors:** Rohit Gaurav, Againdra K Bewtra, Devendra K Agrawal

**Affiliations:** Center of Clinical and Translational Sciences and Department of Biomedical Sciences, Creighton University School of MedicineOmaha, Nebraska

**Keywords:** Airway inflammation, airway remodeling, allergic asthma, chloride channel-3, eosinophils, transcript variants

## Abstract

Eosinophilia is a characteristic feature of allergic airway inflammation and remodeling. Chloride channel-3 (CLC3) in eosinophils has been associated with superoxide generation and respiratory burst. The CLC3 gene may produce multiple transcript variants through alternative splicing. However, the presence of CLC3 variants in human eosinophils is unknown. We examined the expression of CLC3 transcript variants in peripheral blood eosinophils of allergic asthmatics and healthy individuals. Potential of these obligatory dimers to form homo- or hetero-dimers was examined in HEK293 cells co-transfected with CLC3b-GFP and CLC3e-RFP. Eosinophils were isolated from peripheral blood by negative selection. Expression of CLC3 and CLC3 transcript variants was examined by qPCR, Western blot, and immunofluorescence. Confocal micrographs were analyzed with Image J software. Higher levels of novel transcript variants of CLC3 (CLC3b and CLC3e) were found in peripheral blood eosinophils of asthmatics compared to healthy non-atopic subjects. We also found higher CLC3 protein expression in the blood and nasal lavage eosinophils of asthmatics than healthy subjects. Both membranous and intracellular CLC3 expression were observed. Also, we found the presence of both homodimers and heterodimers of CLC3b-GFP and CLC3e-RFP in HEK293 cells. Higher and differential expression of novel CLC3 transcript variants in mild-to-moderate and moderate-to-severe asthmatic eosinophils suggest their critical role in allergic asthma. Membranous and intracellular (granular) expression of CLC3 in nasal lavage and peripheral blood eosinophils suggest their involvement in the activation and migration of eosinophils in allergic asthma. Moreover, homo- and hetero-dimerization of these transcript variants may change the channel properties to exhibit these states. Presence of CLC3 variants may serve as a biomarker in allergic asthma and additional knowledge of interaction between CLC3 transcript variants and their specific role in the activation and migration of eosinophils will allow to explore novel therapeutic approach in allergic asthma.

## Introduction

Eosinophils are pro-inflammatory cells, which migrate to the lungs to cause excessive damage and repair to the airways and result in the exacerbation of asthma 1. The current literature defines eosinophils as innate immunity cells that are classically associated with type I immune response as initiators 2 as well as effectors. However, certain reports question this dogma and suggest that accumulating tissue eosinophils are essentially governors of **L**ocal **I**mmunity **A**nd/or **R**emodeling/**R**epair (LIAR) in healthy as well as diseased condition 3. Studies on human asthmatic patients have shown that eosinophil numbers are significantly increased in bronchoalveolar lavage (BAL) fluid, sputum, endobronchial biopsies in response to airway hyperresponsiveness 4. The underlying molecular mechanism of their migration to the site of inflammation is still unclear.

Chloride-conducting ion-channels, especially the CLC family has emerged as prime contributors to multiple biological processes, including growth, apoptosis, differentiation stabilization of membrane potential, excitation, cell volume regulation, protein degradation, fluid transport, and cell migration 5–8. Expression of CLC3 is reported in multiple cell organelles including lysosomes, cell membrane, and nucleus 9. CLC3 contributes in the acidification of intracellular vesicles 10 and in the loading of transmitters in GABAergic synaptic vesicles in addition to regulating cell volume, migration, and proliferation 11. In addition to smooth muscle cell activation and neointima formation 12, CLC3 is involved in neutrophil oxidative function, phagocytosis, shape change, and migration 13,14. CLC3 inhibits Ca^2+^-activated chloride conductance in epithelial cells 15 modulating epithelial transport and endosomal pH regulation 16. Involvement of CLC3 in the respiratory burst of eosinophils has been documented 17. However, the underlying molecular pathways of eosinophil activation and involvement of CLC3 is not clearly understood.

Like other CLC channels, CLC3 exists as a dimer in a double-barreled configuration 18–20. On alternate splicing, CLC3 gene gives rise to multiple transcript variants. Although, they are believed to form homodimers 21, presence of multiple transcript variants increases the probability of them combining together to form heterodimer as well. Oligomerization in normal and pathological conditions may change the channel properties accordingly. Identifying significance of these transcript variants in specific diseased condition may prove instrumental in understanding the molecular basis of the disease and help in devising therapeutic approaches. Two transcript variants of CLC3 have been relatively well characterized in human genome, CLC3b and CLC3e 22–24. Very little is known about the function of the long form of CLC3 (CLC3e), which is predominantly found on the membrane, co-localized with an epithelial-specific scaffolding protein EBP50 and cytoplasm of epithelial cells 24. CLC3e has an extra 76 bp exon, making its cytoplasmic domain 48 amino acids longer than CLC3b, and has a PDZ-binding domain 23. Short isoform of CLC3 (CLC3b) contains a 202 amino acid long cytosolic C-terminus and has been show to function as a volume-sensitive outwardly rectifying anion channel (VSOAC) in certain cell types 22,25. Hypotonic activation of CLC3b facilitates cell swelling-mediated remodeling of the actin cytoskeleton. Cytosolic C-terminus of CLC3b directly binds to filamentous actin (F-actin) at proline 688 and leucine 734, but not to globular monomeric actin (G-actin) 22,25. Upon CLC3 activation, the direct binding to actin may lead to cytoskeletal rearrangement and migration of eosinophils.

In this study, we examined the presence of novel CLC3 transcript variants in human peripheral blood eosinophils and compared them between healthy subjects and asthmatics to understand the expression profile and relationship with allergic asthma.

## Materials and Methods

### Recruitment of human volunteers

This study was approved by the Institutional Review Board (IRB) of Creighton University. Informed consent was taken from male and female volunteers aged 19–65 years to draw their blood and take nasal washing. Inclusion criteria included refraining from medications including antihistamines for at least 3 days, astemizole for at least 3 months, inhaled β-agonists for at least 8 h, long acting β-agonists for at least 2 days inhaled corticosteroids, nedocromil/cromolyn, or theophylline for at least 2 weeks. Volunteers were asked to refrain from caffeine-containing products for at least 2 days before the nasal washing or blood draw.

Healthy volunteers included subjects without any history of atopy or allergies, and had no illness at the time of recruitment. Volunteers were identified as asthmatics if they had >12% improvement in their lung function, as measured by forced expiratory volume in 1 s (FEV_1_), after inhaled albuterol according to the standard procedure in the Allergy Division at Creighton University Medical Center. According to the standard classification, mild asthmatics have ≥80% of predicted FEV_1_, moderate asthmatics have >60%, but <80% FEV_1_ of predicted FEV_1,_ and severe asthmatics have <60% of the predicted FEV_1_. In this study, mild-to-moderate (M/M) asthma classification included patients with ≥70% FEV_1_ and qualified according to the inclusion criteria. Moderate-to-severe asthmatic (M/S) volunteers had FEV_1_ < 70% of the predicted FEV_1_, and were taking their prescribed Advair Diskus (containing 250/50 or 500/50 mcg of fluticasone propionate and salmeterol, respectively) every day. Due to the health concerns, M/S asthmatics were still on their asthma control medicine at the time of collecting blood and nasal lavage (Table1).

**Table 1:** Information on the recruited volunteers for expression analysis

Volunteers/parameters	Healthy	Mild–moderate asthmatics	Moderate–severe asthmatics
Age (years)	21–62	19–54	30–63
Sex (M/F)	5/5	0/10	5/5
Atopy	No	Yes	Yes
Symptoms	None	>2 days/week but not daily	Daily
Lung function	Normal	FEV_1_ ≥ 70%	FEV_1_ < 70%
		FEV_1_/FVC normal	FEV_1_/FVC reduced ≥5%
Medications (at the time of collecting blood or nasal lavage)	None	None	Advair 250/50 or 500/50

### Isolation of human blood eosinophils

Eosinophils were isolated from the peripheral blood of healthy and asthmatic individuals. Venous blood (120 mL) was collected with EDTA and mixed with HBSS in a 1:1 ratio. Separation of the granulocytes and erythrocytes from the mononuclear cells was done by density gradient centrifugation with Histopaque 1077 (Sigma–Aldrich, St. Louis, MO). Dextran sedimention and hypotonic lysis were used to remove red blood cells. Eosinophils were purified using negative selection in a cocktail of antibodies provided in Eosinophil Isolation Kit (Miltenyi Biotec, Auburn, CA), with autoMACS (Miltenyi Biotec, Auburn, CA). Purity (>99%) and viability (>98%) of eosinophils were determined by staining with Hema-Diff (StatLab, Lewisville, TX), and Trypan Blue (Sigma–Aldrich), respectively.

### Nasal washing

Asthmatic patients were asked to do washing of both the nostrils 3 times each with sinus rinse bottle (NeilMed, Santa Rosa, CA) containing warm (37°C) normal saline. The wash liquid was collected in a sterile beaker. The wash liquid was then filtered with 100 µm filter (Corning) before centrifuging at 300*g* for 5 min at 4°C. Pelleted down cells were washed with HBSS before being fixed in a cytospin slide.

### RNA isolation and reverse transcription

Eosinophils were washed with ice cold PBS and total RNA was isolated using Ambion® mirVana™ miRNA Isolation Kit (Life Technologies, Grand Island, NY) as per manufacturer's instructions. Total RNA (500 ng) was reverse transcribed with ImProm-II™ Reverse Transcription System (Promega, Madison, WI).

### Real-time PCR

Real-time PCR was done using primers for CLC3 TTTATGCCATGGTTGGTGCTGCTG (sense) and ATAAATGCCTTCCCTGCCAAAGGC (antisense), CLC3b CGTTGGCAGTTCTCGGGTGTGTTT (sense) and TATAATGCCAAGGAGGCGCCCAT (antisense), CLC3e TCGAGCAACTAAAGCAGCACGTCG (sense) and TTGGTTTGCCGTCTGGGCCATA (antisense) in CFX96 Real time PCR system (Bio-Rad, Hercules, CA). iQ SYBR Green Supermix (Bio-Rad) was used as the reaction mixture as per manufacturer's instructions. Analysis was done relative to 18S TCAACTTTCGATGGTAGTCGCCGT (sense) TCCTTGGATGTGGTAGCCGTTTCT (antisense) or GAPDH 5′-TCGACAGTCAGCCGCATCTTCTTT-3′ (sense) 5′-ACCAAATCCGTTGACTCCGACCTT-3′ (antisense) as the reference gene in which the value of 2^−ΔΔcq^ was calculated for the fold change in the gene expression in eosinophils isolated from healthy individuals as compared to the asthmatics, where ΔΔCq is (Cq_asthmatic_ − Cq_healthy_)Cq_CLC3_ − (Cq_asthmatic_ − Cq_healthy_)Cq_18S_.

### Protein isolation and Western blot

Freshly isolated eosinophils were washed with ice-cold PBS before lysing them in RIPA buffer (Sigma–Aldrich) and cocktail of protease inhibitors (Sigma–Aldrich) at 4°C. Supernatant was carefully collected in a fresh pre-chilled microcentrifuge tube and kept at −80°C until use. The proteins were transferred from polyacrylamide gels (Bio-Rad) onto nitrocellulose membrane in a standard procedure used in our lab. The membranes were incubated with CLC3 monoclonal antibody (NeuroMab, UCDavis, CA) at dilutions of 1:500 overnight at 4°C in blocking solution, after blocking them in 5% non-fatty blocking grade milk (Bio-Rad) in PBST (Phosphate-buffered saline Tween 20) for an hour. Horse radish peroxidase (HRP)-conjugated anti-mouse secondary antibody at 1:1000 dilution was used in blocking solution. Membranes were washed before visualizing the protein bands under imaging machine (Bio-Rad) with super west dura (Thermo Fisher). Densitometric analysis was performed with ImageJ software (NIH).

### Immunofluorescence and confocal imaging

Pure eosinophils isolated from the blood of healthy and asthmatic individuals and cells collected from the nasal washing of asthmatic patients were spun down in cytospin to fix them onto glass slides. Hydrophobic pen was used to encircle the cells in a small radius compartments. Eosinophils were permeabilized with 4% formalin in PBS for 10 min followed by five thorough washes with PBS for 30 min. Blocking was done with 2.5% goat serum in PBS (blocking solution) for an hour at room temperature. Cells were stained with CLC3 antibodies (GenWay Biotech, Inc., San Diego, CA; NeuroMab) at dilutions ranging from 1:500 to 1:100, overnight at 4°C in 10% blocking solution. Goat anti-rabbit or goat anti-mouse conjugated to Alexa Fluor 488 (Life Technologies) secondary antibodies were used at a dilution of 1:500 for overnight at 4°C in 10% blocking solution after washing the slides 5 times with PBS for an hour at room temperature. Slides were mounted with VECTASHIELD mounting medium containing DAPI (Vector Laboratories, Burlingame, CA), and sealed with nail hardner before keeping them at 4°C in the dark. Confocal microscopy was done in the Integrated Biomedical Imaging Facility of Creighton University School of Medicine. Image analysis was done with ImageJ software (NIH) by calculating the mean fluorescence intensity.

### CLC3 plasmids and HEK293 cell transfection

To examine the homo- or hetero-dimerization of CLC3 protein, HEK293 cells were transfected with plasmids containing CLC3b and CLC3e plasmids. GFP-tagged ORF gene clone of CLC3b (Accession number: NM_001829.2) and RFP-tagged ORF clone of CLC3e (Accession number: NM_173872.2) were obtained from Origene (Rockville, MD). These ampicillin-resistant plasmids were transformed into DH5α ultracompetent cells and amplified. Plasmid isolation was done with PureYield™ Plasmid Mini/Midiprep System (Promega). Fugene HD (Promega) was used to transfect 4 µg of individual plasmids (CLC3b or CLC3e) and 2 µg of each one of them in combination (CLC3b + CLC3e), in a DMEM (Sigma–Aldrich) complete medium (DMEM + 10% FBS) at a ratio of 8:1 for 72 h at 37°C with 5% CO_2_ in HEK293 cells as per manufacturer's instruction. Confocal microcroscopy was performed in the Integrated Biomedical Imaging Core Facility (IBIF) of Creighton University. Cells were visualized under confocal microscope and a Z-stack was taken to check the location and co-localization of these proteins.

To confirm the production of correct mRNA transcripts in the transfected cells, real-time PCR was done with the primers specifically designed to identify respective CLC3 transcript variants, as described above with eosinophils.

### Statistical analyses

One-way ANOVA analyses with Tukey post hoc tests were conducted for all the samples. For skewing the data, an outlier from the healthy group of CLC3 RNA analysis was removed. Due to heterogeneity between-group variances, a Kruskal–Wallis test with Bonferroni-adjusted Mann–Whitney post hoc tests was conducted for CLC3e. For CLC3 protein, mild-to-moderate and moderate-to-severe groups were compared to healthy participants using independent Student's *t*-test.

## Results

### Expression of CLC3 isoforms in asthmatic blood eosinophils

**Figure 1:**
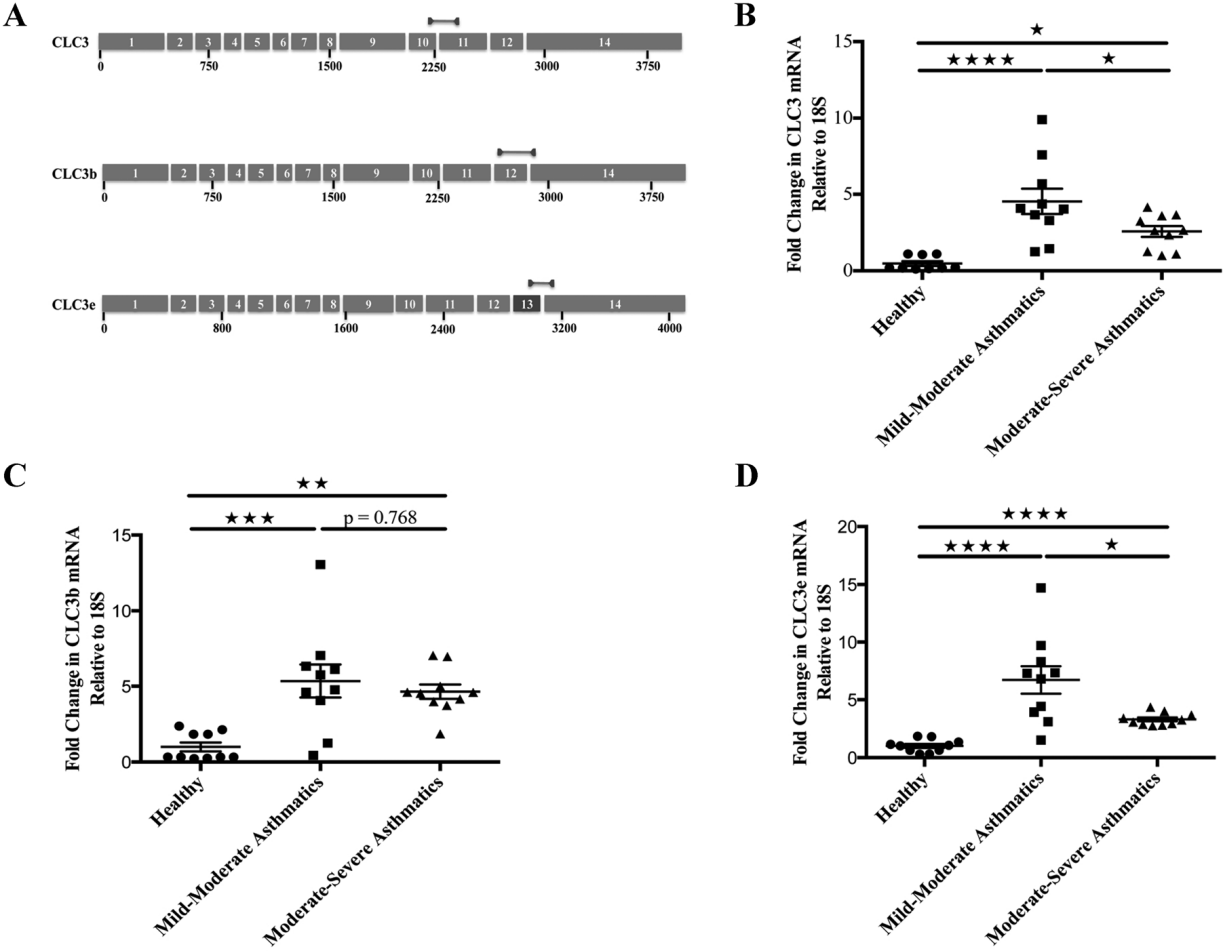
(A) Constructs of the respective primer pairs to identify CLC3 transcript variants. (B)–(D) showing fold change in the mRNA levels of CLC3, CLC3b, and CLC3e, respectively among healthy (*N* = 10), mild-to-moderate (*N* = 10), and moderate-to-severe (*N* = 10) asthmatic peripheral blood eosinophils. *P < 0.05, **P < 0.01, ***P < 0.001, ****P < 0.0001.

We found higher levels of novel transcript variants of CLC3, CLC3b (Primer pair: Exon 12–14 junction, >5-fold) and CLC3e (Primer pair: Exon 13–14 junction, >6-fold), in M/M asthmatic eosinophils compared to eosinophils from healthy individuals (Fig. 1C and D). The CLC3b and CLC3e mRNA levels were significantly higher in M/S asthmatic eosinophils (>4.5-fold and >3-fold, respectively) compared to eosinophils from healthy subjects. Between the asthmatic groups, we found significant reduction in the mRNA level of CLC3e from M/M to M/S asthmatic eosinophils. However, there was no significant change in the level of CLC3b mRNA in M/S asthmatics compared to M/M asthmatic eosinophils. Since M/S patients were on corticosteroids, we checked the effect of corticosteroid on CLC3 transcript variants in eosinophils under in vitro conditions. There was no significant effect of dexamethasone on mRNA transcript levels of CLC3 variants (Suppl. Fig. 1).

We also checked expression of CLC3 with a primer pair at exon 10–11 junction to bind to both the transcript variants of CLC3. Similar to CLC3b and CLC3e transcript variants, CLC3 mRNA level was >4-fold-higher in M/M and >2.5-fold in M/S asthmatics compared to eosinophils from healthy subjects. However, the CLC3 mRNA transcript level did not add up to the other variants. This could be due to either differential binding of the primers used to examine total CLC3 mRNA transcripts versus the binding of primers for individual transcript variants or suggest the possibility of additional transcript variant. Exploring expression and significance of these transcripts may provide another dimension to the physiological and pathophysiological role of CLC3 channels in eosinophils and allergic airway inflammation.

### Expression of CLC3 on the membrane and intracellular organelles of human blood eosinophils

**Figure 2:**
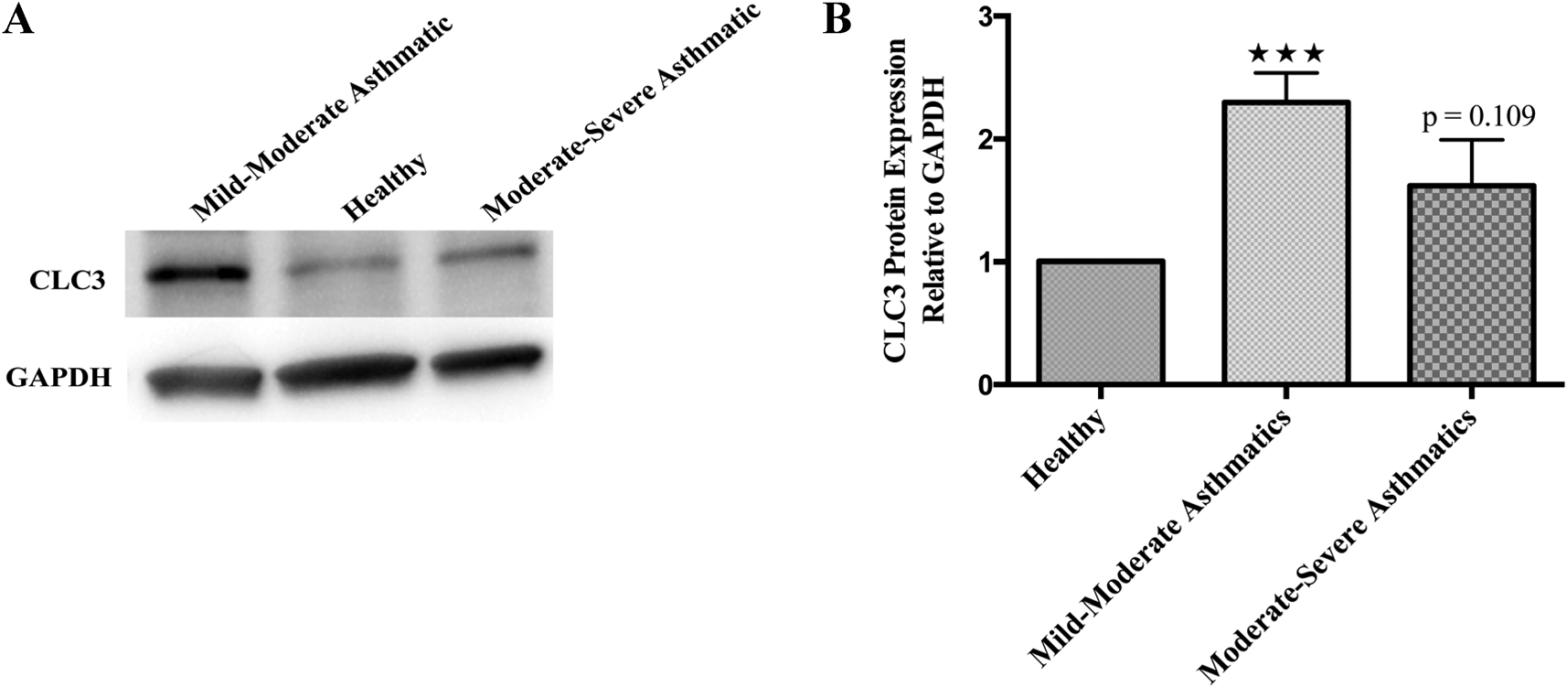
Comparison of CLC3 protein expression among healthy (*N* = 5), mild-to-moderate (*N* = 5), and moderate-to-severe (*N* = 5) asthmatic peripheral blood eosinophils. (A) Western blot showing specific bands for CLC3 protein. (B) Densitometry of Western blot to analyze the relative protein expression. ***P < 0.001.

**Figure 3:**
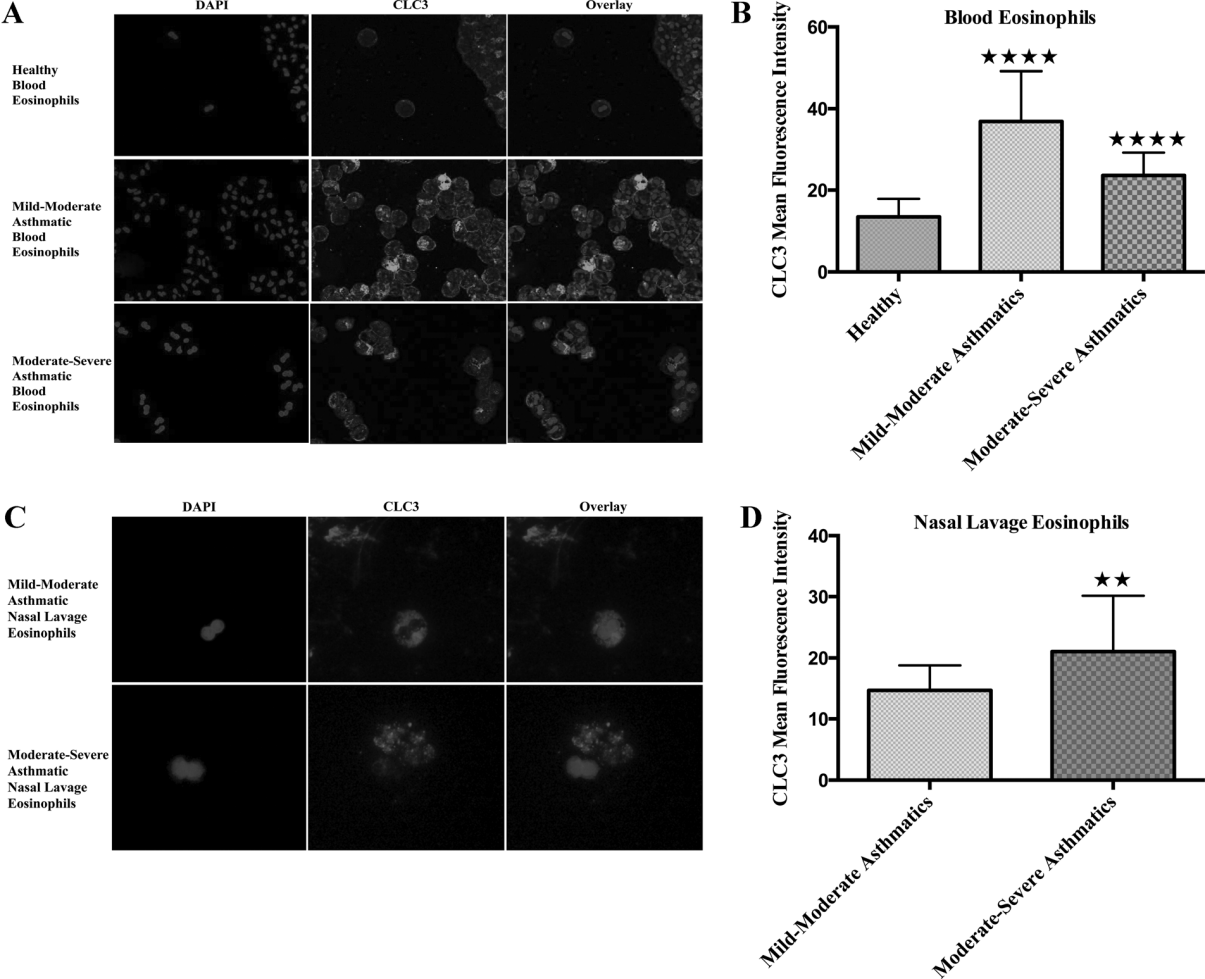
Cellular expression of CLC3 in blood and nasal lavage eosinophils. CLC3 protein expression was significantly higher in M/M (*N* = 5) and M/S asthmatic blood eosinophils (*N* = 5) compared to the healthy subjects (*N* = 5). (A) Confocal micrographs showing membrane and intracellular expression of CLC3 in peripheral blood eosinophils. (B) Image analysis of the confocal micrographs showing higher expression of CLC3 in asthmatic patients. (C) Immunofluorescence micrographs showing intracellular expression of CLC3. The nasal lavage eosinophils in M/S group showed loss of membrane and change in cell morphology indicating degranulation. (D) M/S asthmatic nasal lavage eosinophils (*N* = 4) had higher level of CLC3 compared to M/M asthmatic nasal lavage eosinophils (*N* = 4). **P < 0.01, ****P < 0.0001. To exclude any bias, mean fluorescence of all the cells in the micrographs in the respective slides were analyzed.

To take an active part in the migration of eosinophils, CLC3 should be present on the plasma membrane. Staining with CLC3 antibody showed expression of CLC3 on the plasma membrane of freshly isolated human peripheral blood eosinophils. Protein expression analysis through Western blot exhibited similar trend with highest CLC3 expression in M/M and non-significant difference in M/S asthmatic blood eosinophils compared to healthy individuals (Fig. 2). However, immunofluorescence exhibited lower, but significant, expression of CLC3 in the eosinophils of M/S asthmatics. Confocal micrographs of blood eosinophils showed intracellular and membrane-bound expression of CLC3 with no change in cell size (Fig. 3). But, blood eosinophils, especially from asthmatics, showed heterogeneity in regards to the expression of CLC3 in the cell population, suggesting the presence of dual/multiple phenotype of eosinophils perhaps related to their hypo- and normo-dense characteristics 26,27. We propose that the expression of CLC3 could be a biomarker of the active state of eosinophils in allergic asthma.

### Expression of CLC3 on nasal lavage eosinophils

To correlate our findings in asthmatic blood eosinophils with migrated tissue-bound eosinophils, we examined the expression of CLC3 in the nasal lavage of asthmatic individuals. We were not able to find any inflammatory cells in the nasal washings of healthy volunteers. In the nasal lavage of M/M asthmatics, we found predominantly intact eosinophils with high level of CLC3 expression, mostly on the intracellular granular membranes (Fig. 3). To our knowledge, this is the first report to show intracellular expression of CLC3 in human eosinophils. Intriguingly, unlike blood eosinophils, there was higher expression of CLC3, predominantly present intracellularly, in the nasal lavage eosinophils of M/S than M/M asthmatics. Remarkably, most of the nasal lavage eosinophils in M/S asthmatics appeared to be degranulating and cytolytically releasing free eosinophil granules. However, the exact cause and process of the change in cell morphology is not known and remains to be explored. Cell and nucleus size were also increased and the plasma membrane was not visible.

### Transfection and expression of CLC3b and CLC3e

**Figure 4:**
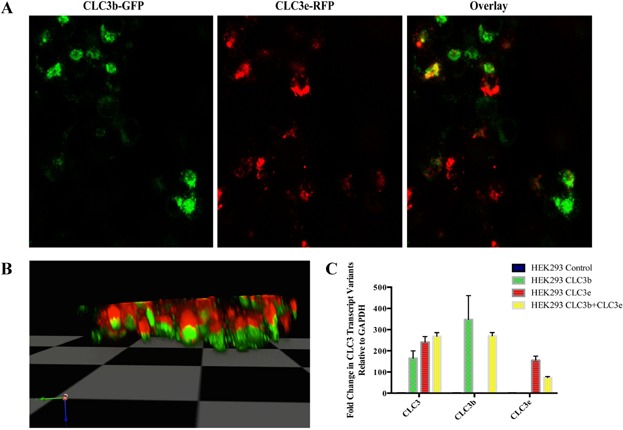
Co-localization of CLC3 transcript variants. HEK 293 cells transfected with CLC3b-GFP and CLC3e-RFP showing homo- and hetero-dimerization. (A) Confocal micrographs showing co-localization of CLC3b-GFP and CLC3e-RFP. (B) Z-stack of the confocal micrograph showing segregation of the transcript variants to apical and basal plane of the cells. (C) qPCR showing change in mRNA levels among respective transfected groups with specific primers designed to detect the CLC3 transcript variants.

Transfection efficiency in HEK293 cells was >90% with individual transcript variants (CLC3b-GFP or CLC3e-RFP) and combination of both (CLC3b-GFP + CLC3e-RFP) plasmids showed plasma membrane as well as intracellular expression of both the proteins. Confocal microscopy showed abundant expression of homo-dimers of CLC3b and CLC3e. However, there was very low degree of co-localization of these proteins, which may suggest a hetero-dimerization (Fig. 4). Interestingly, CLC3b and CLC3e proteins appeared to be compartmentalized in the transfected HEK293 cells, where CLC3e-RFP was segregated on the apical side and CLC3b-GFP was segregated on the dorsal side of the cells (Supplement-2 video).

In order to examine the specific expression of the transcript variants in transfected cells, qPCR with specific primers were performed. PCR amplification confirmed transfection to be isoform-specific in respective groups (Fig. 4).

## Discussion

Increase in CLC3 transcript variants and protein expression in asthmatic eosinophils suggest that the CLC3 variants might play a key role in the migration and activation of these cells in allergic airway inflammation in asthma. Increased CLC3 expression and activity has been related to the migration and activation of neutrophils 13 and to the regulation of respiratory burst in eosinophils 17. Since the protein itself is a dimer, the transcript variants may combine to form homo- or heterodimer (CLC3b-CLC3b, CLC3b-CLC3e, or CLC3e-CLC3e). Different dimer formation and expression localization may modulate the functional characteristics of the cell to make it tuned towards activation or migration pathway. Also, our findings suggest the presence of additional transcript variant(s) of CLC3 in eosinophils that may contribute to their pathophysiological properties. High level of CLC3e transcripts in the eosinophils of M/M asthmatics suggests the key role of CLC3e variant in the migration and activation of eosinophils, and thus involved in the pathophysiology of allergic asthma. However, low level of CLC3 and CLC3e transcripts in M/S asthmatics may be due to inhaled fluticasone propionate, a corticosteroid in Advair 28. Interestingly, CLC3b showed high levels of mRNA transcript even in M/S asthmatics, which may relate to non-responsiveness of this transcript variant to inhaled corticosteroids in asthmatic individuals. Dexamethasone treatment has been shown to reduce CLC3 expression in trabecular meshwork 29. But, in vitro analysis of CLC3 transcripts after dexamethasone treatment of peripheral blood eosinophils did not show any change (Supplement 2). These findings suggest no effect of corticosteroids on CLC3 transcripts. However, the extrapolation of in vitro findings to in vivo could be difficult since the steroid effects in vivo could be cumulative and may modulate multiple molecules in various cell types to induce direct or indirect effects. Inhaled corticosteroids have to go through ADME (absorption, distribution, metabolism, and excretion) after taken up by a patient. Repetitive dose of the drug keeps the corticosteroids in the system for days to have their effect on inflammatory cells and inflammation. However, in our test system, we cannot keep the eosinophils alive for such a long time in vitro. This could explain why we could not see any change in the CLC3 expression with dexamethasone in vitro. None-the-less, additional controlled studies are warranted to confirm this finding.

Consistent with the blood eosinophils, we found reduced number of eosinophils in the nasal lavage of M/S patients on inhaled corticosteroids. However, unlike blood eosinophils, CLC3 expression was more in M/S nasal lavage eosinophils, possibly due to the increased expression of CLC3 intracellularly. Expression of CLC3 in the lysosomes is required for its acidification 10,30. Increased intracellular expression of CLC3 may relate to the activated state and degranulation of eosinophils. Blood eosinophils showed a membrane-bound and intracellular expression of CLC3, but the nasal lavage eosinophils in asthmatics showed predominantly intracellular expression of CLC3. Eosinophil granules may function as extracellular receptor-mediated secretory organelles 31, and expression of CLC3 on eosinophil granules might regulate their activity. More studies are required to precisely locate the intracellular expression of CLC3 in eosinophils and its significance in the intracellular compartments.

Co-transfection experiments on HEK293 cells with CLC3b-GFP and CLC3-RFP plasmids suggest homo- and hetero-dimerization of these transcript variants. However, the level of hetero-dimerization was extremely low. It is likely that the basal expression of these transcript variants is essentially homo-dimer, and hetero-dimerization only occurs in the activated state of cells under inflammatory cytokine milieu. This hypothesis is yet to be tested. Although perplexing, the segregation of CLC3e-RFP on apical side and CLC3b-GFP on the distal side of HEK293 cells could relate to protein localization for migration of cells.

The expression pattern of CLC3 suggests its importance in the migration of blood eosinophils, and in activation and degranulation of nasal lavage eosinophils in allergic asthma. Novel finding of CLC3 transcript variants in human blood eosinophils may open doors for further identification and involvement of new transcript variants of CLC3 in allergic asthma, and thus in the development of novel therapeutic approaches.

Understanding the underlying signaling events taking place in the activation and migration of eosinophils through CLC3 will help us to understand the complex interactions of these proteins in these hyperactivated cells in allergic asthma. The information from such studies would provide an opportunity to develop novel therapeutic approaches in allergic asthma.
