# Supplementary figures and images for: Novel CLC3 transcript variants in blood eosinophils and increased CLC3 expression in nasal lavage and blood eosinophils of asthmatics
Source: Immun Inflamm Dis. 2014 Dec 4;2(4):205–13. doi: 10.1002/iid3.36 (PMC4386915; doi:10.1002/iid3.36)

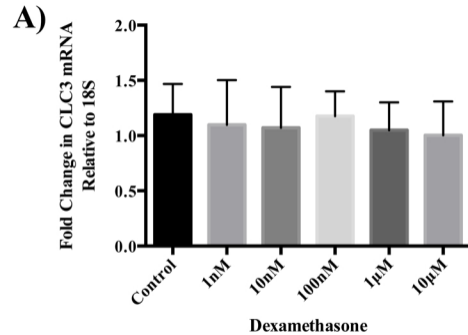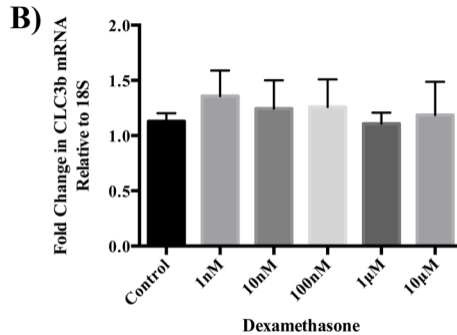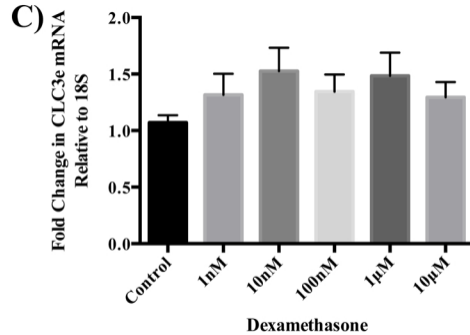

Supplement: Supplementary file 1 [file iid30002-0205-sd1.pdf]

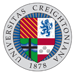

Supplement: Supplementary file 2 [file iid30002-0205-sd2.gif]
